# Fluid Structural Analysis of Urine Flow in a Stented Ureter

**DOI:** 10.1155/2016/5710798

**Published:** 2016-03-31

**Authors:** J. Carlos Gómez-Blanco, F. Javier Martínez-Reina, Domingo Cruz, J. Blas Pagador, Francisco M. Sánchez-Margallo, Federico Soria

**Affiliations:** ^1^Jesús Usón Minimally Invasive Surgery Centre, Carretera N-521, km 41.8, 10071 Cáceres, Spain; ^2^Department of Mechanical Engineering, Universidad de Sevilla, C∖Camino Descubrimientos, S/N, Isla de la Cartuja, 41092 Sevilla, Spain

## Abstract

Many urologists are currently studying new designs of ureteral stents to improve the quality of their operations and the subsequent recovery of the patient. In order to help during this design process, many computational models have been developed to simulate the behaviour of different biological tissues and provide a realistic computational environment to evaluate the stents. However, due to the high complexity of the involved tissues, they usually introduce simplifications to make these models less computationally demanding. In this study, the interaction between urine flow and a double-J stented ureter with a simplified geometry has been analysed. The Fluid-Structure Interaction (FSI) of urine and the ureteral wall was studied using three models for the solid domain: Mooney-Rivlin, Yeoh, and Ogden. The ureter was assumed to be quasi-incompressible and isotropic. Data obtained in previous studies from ex vivo and in vivo mechanical characterization of different ureters were used to fit the mentioned models. The results show that the interaction between the stented ureter and urine is negligible. Therefore, we can conclude that this type of models does not need to include the FSI and could be solved quite accurately assuming that the ureter is a rigid body and, thus, using the more simple Computational Fluid Dynamics (CFD) approach.

## 1. Introduction

Urine flows from the renal pelvis to the urinary bladder in healthy people through the ureter for two reasons: a physiological activity (peristalsis) and a physical phenomenon (pressure gradient in an open system). Some common pathologies that can block ureters are stones, inflammations, or tumorous tissues. These problems can reduce or even stop the urine flow and provoke more serious pathologies and risks for patients.

In order to solve this problem, two different treatments are usually performed: (1) a nephrostomy that temporarily opens the urinary tract using an external tube and (2) a ureteral stenting that inserts a flexible tube named stent into the ureter. Both techniques achieve restoring the urine flow, decreasing intrapelvic pressure, and avoiding a renal failure. In those cases where a stent is used in blocked ureters, this stent causes a gradual loss of muscle tone [1, 2] and the ureter does not produce the peristaltic activity anymore. Hence, stented ureters are only influenced by the pressure gradient.

Currently, all these treatments are tested and tuned up using experimental models that allow mimicking quite accurately the physiological behaviour under different boundary conditions. The pig is believed to be the ideal model for urologic and endourologic research, because it has the renal and ureteral anatomy and physiology similar to humans [3]. However, some studies are difficult or impossible to be performed with clinical experiments for different reasons: measurement constraints, ethical considerations, instrumental limitations, and so forth. For this reason, finite element (FE) models and other computational models have been introduced as a simulation tool of the urine flow and for assisting in the design and development of new ureteral stents, among others [4–6].

Previous studies have conducted several experiments to obtain the mechanical behaviour of the ureteral wall. Yin and Fung [7] performed different ex vivo tests to obtain the strain-stress curves of a ureter and define its mechanical properties. Later, in vivo characterizations were performed by Sokolis [8] showing the anisotropic behaviour of ureters, which was modelled with a four-parameter Fung-type strain energy function. More recently, Rassoli et al. [9] provided valuable data of human ureter strain-stress curves fitted with a four-parameter Fung-type model and five-parameter polynomial models.

On the other hand, Computational Fluid Dynamics models have been used by Lozano [10] or Vahidi [11, 12] to simulate peristaltic movements of a ureter in a 2D analysis. Later, Hosseini et al. [13] extended that analysis to the 3D case using FE for the CFD calculation. Other authors [14–16] concentrated their efforts on the analysis of the urine movement in a stented ureter. The background just described analyses the mechanical behaviour of a ureter and the movement of urine during peristalsis or in a stented ureter. All the approaches to study urine movement, except in Hosseini's work, were axisymmetrical CFD analyses and most of them were about peristalsis. Hosseini analysed the 3D movement, but reproducing the peristaltic movement not the flow through a stented ureter. There are no previous studies that focus its interest on the possible influence of a stented ureter in the urine flow.

Hence, the objectives of this work are (1) to define an experiment that allows obtaining real pressure data, used as boundaries conditions, in an animal model (2) and to check through 3D Fluid-Structure Interaction (FSI) if stresses and strains in a stented ureter justify the need of using FSI simulations between urine flow and a stented ureter.

## 2. Material and Methods

It is well known that the ureteral wall is a soft tissue reinforced with collagen fibres [17, 18]. The fibre arrangement is highly directional as proved by Sokolis, who confirmed experimentally that the mechanical behaviour is more properly captured using an anisotropic model, for example, using a four-parameter Fung-type strain energy function [8]. However, in a more recent study, this author has confirmed that the fibre directionality is different for the three layers of the ureteral wall (tunica mucosa, tunica adventitia, and tunica muscularis) [19] and variable along the length of the ureter, being the lower ureter less anisotropic than the upper ureter. Nothing is known about the directionality of fibres in a stented ureter in which the ureteral walls are stretched and the fibres are likely reoriented. For this reason and for the sake of simplicity, the ureteral wall has been modelled as an isotropic hyperelastic quasi-incompressible material. Some experimental data of stress-strain curves of the ureteral wall found in the literature [8, 9, 11, 14] have been fitted to four models: neo-Hookean, Mooney-Rivlin, Yeoh, and one-term Ogden.

### 2.1. Mathematical Models Used

#### 2.1.1. Mooney-Rivlin Model

The strain energy density function is as follows:

(1) $$\begin{array}{c} U=C_{10}\left(\;{\bar{I}}_{1}-3\;\right)+C_{01}\left(\;{\bar{I}}_{2}-3\;\right)+\frac{1}{D_{1}}{\left(\;J-1\;\right)}^{2}, \end{array}$$

where *C*_10_ > 0 and *C*_01_ > 0 are the material constants, while the constant *D*_1_ is chosen a priori, small enough to force the incompressibility. ${\bar{I}}_{1}$ and ${\bar{I}}_{2}$ are the first and second invariant of the modified Cauchy Green tensors and *J* is the volume ratio.

#### 2.1.2. Neo-Hookean Model

The strain energy density function is as follows:

(2) $$\begin{array}{c} U=C_{10}\left(\;{\bar{I}}_{1}-3\;\right)+\frac{1}{D_{1}}{\left(\;J-1\;\right)}^{2}. \end{array}$$

Neo-Hookean model can be seen as a particular case of the Mooney-Rivlin model in which *C*_01_ = 0.

#### 2.1.3. Yeoh Model

The strain energy density function is as follows:

(3) U=C1I¯1−3+C2I¯1−3+C3I¯1−3+1D1J−12+1D2J−14+1D3J−16,

where *C*_*i*_ are the three material constants and *D*_*i*_ are chosen to force incompressibility. Typically, *C*_1_ > 0, *C*_2_ < 0, and *C*_3_ > 0.

#### 2.1.4. One-Term Ogden Model

The strain energy density function is as follows:

(4) $$\begin{array}{c} U=\frac{2\mu_{p}}{\alpha_{p}^{2}}\left(\;{\bar{\lambda }}_{1}^{\alpha_{p}}+{\bar{\lambda }}_{2}^{\alpha_{p}}+{\bar{\lambda }}_{3}^{\alpha_{p}}-3\;\right)+\frac{1}{D_{1}}{\left(\;J-1\;\right)}^{2}, \end{array}$$

where ${\bar{\lambda }}_{i}$ are the modified principal stretches, *μ*_*p*_ and *α*_*p*_ are the material constants, and *D*_1_ is chosen to force incompressibility.

### 2.2. Curve Fitting of the Behaviour Curves

The mechanical behaviour of ten different human ureters was studied by Rassoli et al. [9] through biaxial tests, aimed at obtaining both longitudinal and circumferential stresses and deformations of those ureters. Those tests were fitted in the present work using the models described above and the Levenberg-Marquardt algorithm. From the ten original specimens, five were selected as they had the behaviour most similar to isotropic (similarity between the longitudinal and circumferential curves provided by Rassoli et al.) and, from them, only three gave rise to model constants that were thermodynamically consistent. Those were the three specimens selected for the present study.

The tests selected for fitting were the longitudinal ones, though similar results could be obtained for the circumferential set of tests, given the preselection of the most isotropic specimens commented on above. After analysing the goodness of the fit, the Neo-Hookean model was dismissed since it could not capture reasonably well the behaviour of the tested ureters.

The results of the curve fitting in the longitudinal direction for all the selected ureters are shown in Table 1.

**Figure 1:**
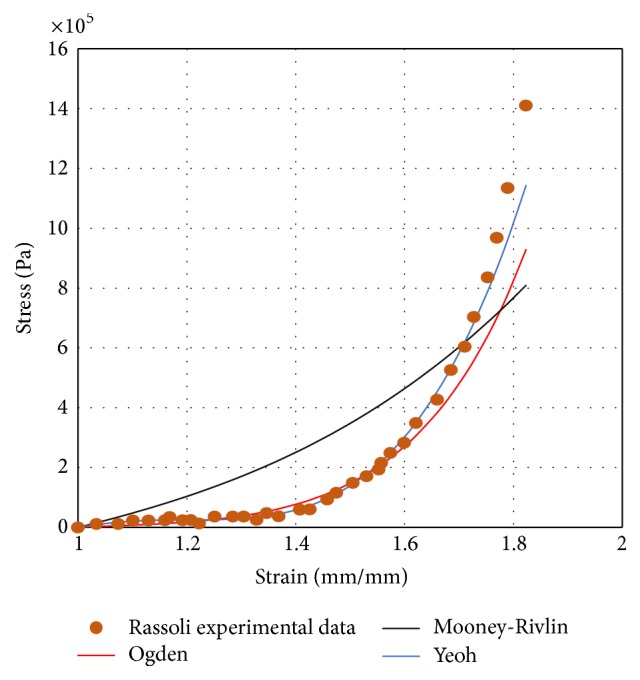
Fitting curves for flexible ureter.

The selected ureters are sorted by stiffness in a descending order from 1 to 3 and will be named from now on stiff, medium, and flexible ureters, respectively. Ogden, Mooney-Rivlin, and Yeoh fitting curves for flexible ureter are shown in Figure 1.

### 2.3. Geometry

The length of a healthy ureter from the Ureteropelvic Junction (UPJ) to the Vesicoureteral Junction (VUJ) is about 28 cm long. In this study, it was assumed that the stented ureter (opened after the muscle tone is lost) is a cylindrical tube of that length and a thickness of 1 mm (assumed to be constant) plus an inner diameter of 3 mm. All these data were measured in an experiment of a swine model.

**Figure 2:**
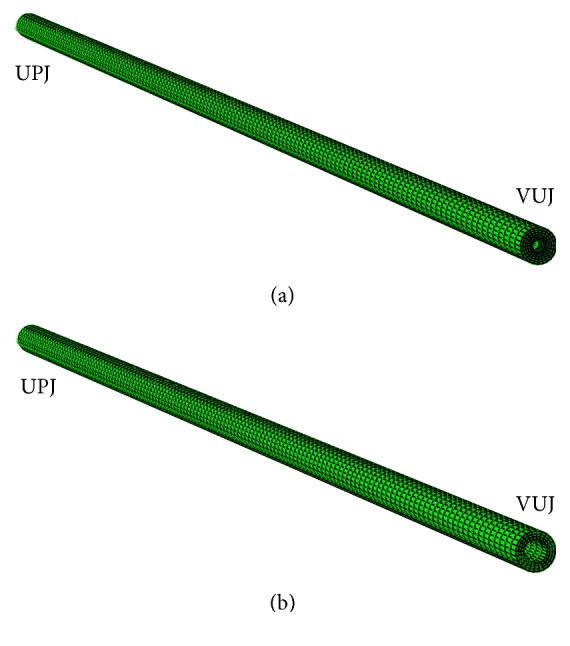
Fluid domain mesh (a) and solid domain mesh (b).

The space inside the ureter was assumed to be filled up with urine, therefore simulating a state of diuresis peak. This fluid domain (Figure 2(a)) was meshed with 16800 hexahedral linear elements for fluid simulations (FC3D8 from the ABAQUS Elements Library). The solid domain representing the ureter (Figure 2(b)) was meshed using 10080 hexahedral linear continuum elements (C3D8H) and the stent was a cylinder coaxially disposed with the ureter and with a diameter of 1 mm. The stent is much stiffer than the rest of materials in the model and thus it was assumed to be a rigid body. These mesh sizes were chosen after performing a convergence analysis and they can be seen in Figure 1.

### 2.4. Models Constitutive Equations

The solid domain is generally governed by the dynamic equilibrium equations:

(5) ρai−σij,j−ρfi=0in  D,

(6) σijnj−ti=0in  ∂D,

where *i* refers to the *i*th component of the equations, *ρ* is the density, *a*_*i*_ is the spatial acceleration, *f*_*i*_ is the body force per unit mass, *σ*_*ij*_ are the components of the Cauchy stress tensor, *n*_*j*_ are the components of the normal unit vector, and *t*_*i*_ are the components of the Cauchy traction vector. The inertial forces are negligible in this problem so that the first term of (5) can be dismissed.

For the fluid domain, urine is considered a Newtonian, incompressible fluid and the flow is assumed to be laminar. The density (993.3 kg/m^3^) and dynamic viscosity (0.6913 cP) of urine [20] were assumed to be equal to those of water at 37°C. The Navier-Stokes equations govern the movement of urine inside the ureter:

(7) ∇·v→=0,ρ∂v→∂t+∇p=μ∇2v→+ρf→,

where $\vec{v}$ is the spatial velocity vector, *ρ* is the fluid density, *p* is the static pressure, *μ* is the dynamic viscosity, and *f* is the body force vector.

The FSI is given by the nonpenetration and nonslippery conditions:

(8) dis=dif,∂dis∂t=∂dif∂t,

where *d*_*i*_^*s*^ and *d*_*i*_^*f*^ are the *i*th component of the solid and fluid domain displacements, respectively, and the equilibrium equations at the fluid-solid interphase are

(9) $$\begin{array}{c} n_{j}\sigma_{ij}^{s}=n_{j}\sigma_{ij}^{f}, \end{array}$$

where the superscripts of the stress tensors refer to solid and fluid, respectively.

### 2.5. Experimental Measurements of Boundary Conditions

The FE model of the ureter was subjected to the following loads: (1) intra-abdominal pressure applied on the outer surface of the ureteral wall by loose connective tissue and the organs surrounding the ureter, (2) UPJ pressure, exerted by urine in the renal pelvis, and (3) VUJ pressure, which is the fluid pressure in the bladder.

Experiments with swine models were done in order to obtain realistic values of those pressures. Two healthy large white breed female pigs with a weight of 35 kg were used. Once these two subjects were anaesthetised with inhalation anaesthesia and the urinary tract was evaluated to detect possible alterations that could have had any influence on the study, both subjects were accepted in the experimental phase. A 7Fr Double-J ureteral stent was bilaterally placed in each of the two ureters of the two subjects.

The minimum lapse of time needed to provoke the passive dilatation of the upper urinary track and the ureterectasis is about a week [1]. Hence, one week after insertion of the stents, the measurements of renal pelvis, urinary bladder, and intra-abdominal pressures were carried out in both subjects. The procedure of intrapelvic pressure measurement was done by a needle connected to a Dräger Infinity Gamma (Germany) pressure system; this renal puncture was done with an eco-guided procedure in both kidneys. Both the ureter length and internal diameter were measured from UPJ to VUJ under C arm (Philips Bv 300, Netherlands) fluoroscopic control. The intravesical pressure was measured with the same pressure system used before. In this case, the measurements were done after draining the bladder through a Foley catheter. Finally, the intra-abdominal pressure was measured with a pressure sensor located in the peritoneal cavity and inserted through a transabdominal percutaneous puncture.

Once the four urinary tracts baseline measurements (repose and fasting measures of organic function) were done, the last step was the instillation. This instillation was performed through intrapelvic percutaneous access of iodinated medium contrast with saline to raise up the UPJ pressure 6 mmHg and stimulate the draining of the urinary bolus though the ureter. During this draining, the pressures specified above were measured five times [21–23] for each ureter obtaining the values shown as follows:

- Ureter length = 28 cm.
- Intra-abdominal pressure = 266–532 Pa.
- UPJ pressure = 265–667 Pa.
- VUJ pressure = 266 Pa.

### 2.6. FSI Simulations

Diuresis peaks were simulated using the cosimulation techniques of Abaqus FEA, which couples the Abaqus/standard and Abaqus/CFD modules. Since the transient period is very short, only 20 seconds were simulated, by imposing the boundaries conditions:

- Intra-abdominal pressure = 533.28 Pa.
- UPJ pressure = 666.61 Pa.
- VUJ pressure = 266.24 Pa.

Nine simulations were done, combining the three models (Mooney-Rivlin, Ogden, and Yeoh) and the three ureters (stiff, medium, and flexible).

## 3. Results and Discussion

The maximum and minimum stresses and strains obtained are shown in Tables 2 and 3 for the flexible ureter, Tables 4 and 5 for the medium, and Tables 6 and 7 for the rigid one. Both stresses and strains are given in cylindrical coordinates.

The stresses are very similar regardless of the model and the ureter and are exclusively controlled by the external pressure. In contrast, the strains are quite different (with a noticeable influence of the model and the ureter) and very small compared to the typical values of a soft tissue.

In general, the strains obtained with the Ogden and Yeoh models are very similar to each other and very different from those obtained with the Mooney-Rivlin model. An exception to this is the flexible ureter, where the strains are one order of magnitude greater with the Ogden model. The reason for this is the ability of the Ogden model to better capture the toe region of the ureteral wall. This makes the Ogden model the most appropriate one to simulate the ureter's behaviour and results in a more flexible behaviour for low values of the stresses, as is the case in these simulations. Obviously, the strains of the stiff ureter are smaller than those of the medium one and these are smaller than in the flexible ureter.

**Figure 3:**
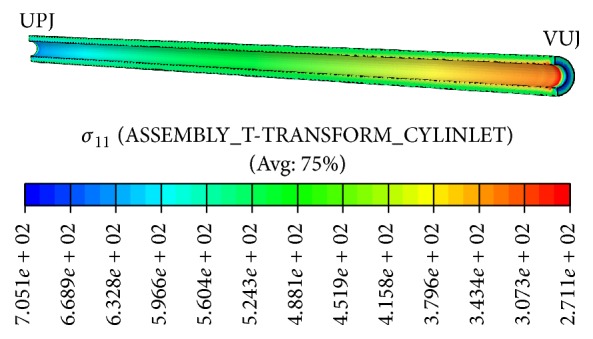
Radial stresses distribution in solid domain.

**Figure 4:**
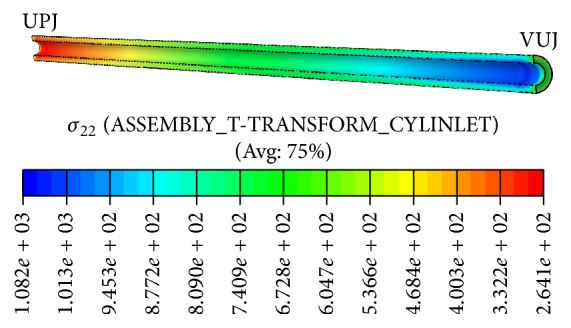
Circumferential stresses distribution in solid domain.

**Figure 5:**
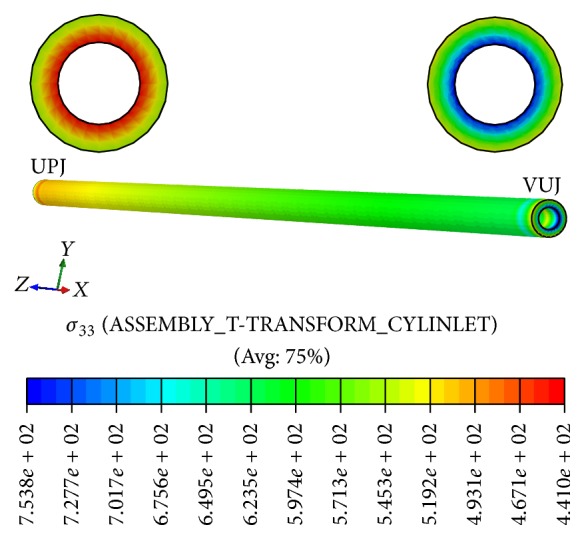
Longitudinal stresses distribution in solid domain.

The distributions of radial, circumferential, and longitudinal stresses in the ureteral walls are shown in Figures 3, 4, and 5, respectively. The radial stress at the interface is equal in both domains due to equilibrium and so its distribution reflects the fluid pressure distribution. These radial stresses are compressive with their maximum absolute value near the UPJ, except for the urine-ureter interface. The radial stresses are very uniform.

Compressive stresses in the circumferential direction (Figure 4) are caused by the pressure difference between the urine and the intra-abdominal space and the finite thickness of the ureteral wall that makes the outer pressure to be exerted over a wider area. So, the compressive circumferential stresses are higher where the intra-abdominal pressure is greater than the urine pressure, in the VUJ.

Longitudinal stresses are also compressive and vary much along the ureter, with its maximum value near the bladder, in VUJ.

### 3.1. Strains

The strains the ureteral wall is subjected to are much lower than the typical values of other soft tissues. This is due to the low pressure exerted by urine during the draining of the renal pelvis, which produces very low stresses and keeps the tissue in the toe region of the stress-strain curve. This fact highlights the necessity of simulating its mechanical behaviour with a model that captures well this toe region, as the Ogden model proved to do. Furthermore, pressure and, consequently, stresses in the ureter are so low that the strains are negligible and the ureter behaves as a rigid solid with this pressure regime.

The assumptions done to characterize the whole system involve some limitations. The use of a perfectly cylindrical ureter is not realistic, since a real ureter is a curved tube that may cause the urine flow to collide against the ureter's inner walls, so increasing the dynamic pressure and thus the strains, along with other perturbations in the pressure and velocity of the stationary urine flow, is not considered here. The variation between urine and water properties, although minimum, has not been considered and could have an important influence on the final results at low flow rates. Furthermore, additional experiments with a greater number of subjects should be performed to obtain more reliable and robust experimental boundary conditions data. Nonetheless, the experiment conducted in this preliminary study, with only two subjects, shows promising results that should be confirmed in future studies.

An important limitation of the study is that the ureter has been assumed to be isotropic and it is well known that it is a fibred material. However, most of the stress-strain curves obtained by Rassoli et al. [9] (and particularly those used in this study) showed a more or less isotropic behaviour, so that this simplification is justified to a certain extent. Moreover, in order to consider the anisotropy in detail, the three layers of the ureteral wall and regional differences along the ureter should be considered as reported by Sokolis [8, 19], therefore complicating the study considerably. In any case, this does not seem worthwhile in light of the level of stresses and strains seen in the ureter. Finally, the effect of submergence on the flow around the stent should be analysed in further detail, for example, following the work by Ikram et al. [24].

## 4. Conclusions

In this study, a numerical simulation of the renal pelvis draining through a stented ureter with a simplified geometry has been analysed with FE and considering the Fluid-Structure Interaction. Several material models have been selected to describe the ureter's mechanical behaviour and different sets of constants were tested to consider the influence of the overall stiffness of the ureter. According to the soft-tissue behaviour of the ureter, it can be expected that the application of small stresses could cause large deformations. However, our results concluded that the behaviour of the ureter during urine flow is nearly the same regardless of the model used and the stiffness of the ureter. Notwithstanding, the best was the Ogden model, since it could capture the toe region of the stress-strain curves provided by Rassoli et al. [9] more accurately.

The stresses produced by the urine and intra-abdominal pressures were very low and the strains almost negligible, so that it can be concluded that the ureter behaves as a rigid structure in this pressure regime and the FSI simulation, which is very demanding from a computational point of view, could be replaced by a simple CFD simulation.

On the other hand, additional efforts should be performed to obtain more realistic models of the urinary tract in future works. First, a perfect cylinder was used to represent the ureter's geometry. In this sense, future works should use more realistic geometric models provided by CT or MRI images. Second, the assumption of the properties of urine as equal to those of water should be corrected by obtaining urine properties in additional experiments. Finally, additional experiments of all these aspects related to the mechanical characterization of the organic tissues will be done in future studies.

## Figures and Tables

**Table 1:** Curve fitting parameter values for stiff (1), medium (2), and flexible (3) ureter.

Ureter	Mooney-Rivlin		Yeoh			Ogden	
	*C*_10_ (kPa)	*C*_01_ (kPa)	*C*_1_ (kPa)	*C*_2_ (kPa)	*C*_3_ (kPa)	*μ*_*P*_ (kPa)	*α*_*P*_
1	3.94	3383.60	802.82	−14.53	1512.65	1699.32	0.13
2	12.21	1796.80	220.97	−16.00	414.23	372.07	0.13
3	0.90	376.09	258.42	−195.00	105.43	168.56	0.10

**Table 2:** Maximum and minimum stresses obtained for the flexible ureter.

Model	*σ*_*r*_ (Pa)		*σ*_*θ*_ (Pa)		*σ*_*Z*_ (Pa)	
	Max	Min	Max	Min	Max	Min
Mooney-Rivlin	−272.08	−676.25	−267.08	−1082.33	−442.50	−738.97
Ogden	−272.36	−721.43	−261.01	−1077.58	−439.10	−762.51
Yeoh	−271.78	−691.80	−265.45	−1082.54	−441.25	−746.95

**Table 3:** Maximum and minimum strains obtained for the flexible ureter.

Model	*ε*_*r*_ (%)		*ε*_*θ*_ (%)		*ε*_*Z*_ (%)	
	Max	Min	Max	Min	Max	Min
Mooney-Rivlin	0.22	−0.13	0.12	−0.28	0.06	−0.04
Ogden	1.06	−0.52	0.60	−1.23	0.29	−0.19
Yeoh	0.33	−0.18	0.18	−0.41	0.09	0.00

**Table 4:** Maximum and minimum stresses obtained for the medium ureter.

Model	*σ*_*r*_ (Pa)		*σ*_*θ*_ (Pa)		*σ*_*Z*_ (Pa)	
	Max	Min	Max	Min	Max	Min
Mooney-Rivlin	−274.04	−663.89	−235.13	−1074.49	−385.45	−679.04
Ogden	−271.11	−705.08	−264.00	−1081.54	−441.01	−753.81
Yeoh	−271.70	−696.74	−264.86	−1082.48	−440.64	−749.55

**Table 5:** Maximum and minimum strains obtained for the medium ureter.

Model	*ε*_*r*_ (%)		*ε*_*θ*_ (%)		*ε*_*Z*_ (%)	
	Max	Min	Max	Min	Max	Min
Mooney-Rivlin	0.032	−0.039	0.020	−0.073	0.013	−0.022
Ogden	0.470	−0.246	0.265	−0.566	0.130	0.084
Yeoh	0.394	−0.210	0.221	−0.476	0.107	−0.071

**Table 6:** Maximum and minimum stresses obtained for the stiff ureter.

Model	*σ*_*r*_ (Pa)		*σ*_*θ*_ (Pa)		*σ*_*Z*_ (Pa)	
	Max	Min	Max	Min	Max	Min
Mooney-Rivlin	−275.55	−664.57	−192.51	−1076.61	−298.70	−627.02
Ogden	−272.78	−663.36	−268.06	−1078.86	−442.68	−713.95
Yeoh	−272.76	−663.38	−269.83	−1079.38	−442.83	−716.00

**Table 7:** Maximum and minimum strains obtained for the stiff ureter.

Model	*ε*_*r*_ (%)		*ε*_*θ*_ (%)		*ε*_*Z*_ (%)	
	Max	Min	Max	Min	Max	Min
Mooney-Rivlin	0.010	−0.027	0.008	−0.046	0.007	−0.017
Ogden	0.087	−0.066	0.050	−0.135	0.028	−0.028
Yeoh	0.094	−0.069	0.053	−0.142	0.030	−0.029
